# Root cause analysis investigation of visible particulates in therapeutic protein drug products using morphologically directed Raman spectroscopy

**DOI:** 10.1038/s41598-025-97097-x

**Published:** 2025-11-26

**Authors:** Isabella F. de Luna, Srivalli N. Telikepalli, Michael Carrier, Dean Ripple, Charudharshini Srinivasan, Thomas O’Connor, Scott Lute, Ashwinkumar Bhirde

**Affiliations:** 1 Division of Pharmaceutical Quality Research VI, Office of Pharmaceutical Quality Research, Office of Pharmaceutical Quality, Center for Drug Evaluation and Research, Food and Drug Administration, Silver Spring, MD, USA; 2 Division of Product Quality Assessment II (DPQAII), Office of Product Quality Assessment (OPQA I), Office of Pharmaceutical Quality, Center for Drug Evaluation and Research, Food and Drug Administration, Silver Spring, MD USA; 3 Biomolecular Measurement Division, National Institute of Standards and Technology, Gaithersburg, MD USA

**Keywords:** Protein aggregation, Principal component analysis, Raman spectroscopy, Monoclonal antibodies, Morphology, Insulin, Particle size, Biopharmaceutical characterization, Imaging methods, Biotechnology, Chemistry, Materials science

## Abstract

Visible particulate (VP) matter in therapeutic protein drug products (DPs) is a quality attribute that can impact product efficacy and patient safety across the product lifecycle. This study evaluates Morphologically Directed Raman Spectroscopy (MDRS) for detecting and characterizing VPs in therapeutic protein DPs. MDRS combines morphological and chemical analyses, which are essential for root cause analysis (RCA) during incident investigations of VP formation. We evaluated MDRS using polystyrene (PS) microsphere standards, candidate reference materials from the US National Institute of Standards and Technology (NIST), and stressed therapeutic protein DPs, insulin lispro and rituximab. Using MDRS, VPs were imaged and chemically characterized in insulin or rituximab drug products with varying morphologies and ranged from 100 to 400 μm in diameter. The intensity of Raman spectral peaks varied with VP size, transparency, morphological complexity, and between therapeutic protein drug classes. Principal component analysis (PCA) of morphological features (e.g., size, shape, and transparency) revealed that NIST ethylene tetrafluoroethylene (ETFE) particles displayed a range of morphologies, akin to stressed therapeutic protein VPs. These results highlight the potential of combining morphological analysis with Raman spectroscopy to support RCA in therapeutic protein DPs for both pre- and post-market quality assessments.

**Supplementary Information:**

The online version contains supplementary material available at 10.1038/s41598-025-97097-x.

## Introduction

Therapeutic protein drug products (DPs) are used to treat a range of conditions, including cancer, immune disorders, diabetes, genetic disorders, and infectious diseases^1^. Evaluating critical quality attributes (CQAs) is essential for ensuring safety and efficacy, and recent innovations in therapeutic protein development, such as increased molecular complexity and novel manufacturing approaches^2^ places an even greater emphasis on the need for CQA assessments throughout the product life cycle. Visible particulate (VP) matter in protein therapeutics are typically CQAs, as the presence of VPs can impact patient safety and product efficacy^3^. Particulates in therapeutic protein drug products have been reported to cause adverse reactions, including granuloma formation, injection site reactions, and blood vessel obstructions, potentially resulting in serious complications like stroke or even death^4,5^. Since 2018, there have been 46 drug recall notices related to VPs, with 28% of recall notices for injectable drug products attributed to this issue^6^.

According to the United States Pharmacopeia (USP) General Chapter, *Particulate Matter in Injections* < 788>, particulates are defined as “mobile, undissolved particles other than gas bubbles that are unintentionally present in an injectable product”^7^. USP *Visible Particulates in Injections < 790 >* further states that due to the potential to jeopardize patient safety, each batch must be “essentially free of visible particulates”, with 100% of finished units inspected prior to release^8^. Adherence to criteria outlined in USP General Chapters on particulate matter is important for manufacturers to fully comply with current good manufacturing (cGMP) standards and ensure safe and efficacious DPs.

Particulates can be classified by source as extrinsic, intrinsic, or inherent^9^. Extrinsic particles originate from foreign contaminants, such as clothing fibers, hair, or skin. Intrinsic particles are associated with the manufacturing process, including materials like glass or polymer fragments. Inherent particles arise from the therapeutic protein or formulation components, with high molecular weight protein aggregates being the most notable. High molecular weight protein aggregates can elicit immune responses in patients^10–12^, making their identification and characterization necessary.

Particles are also classified by size: nanometer (< 100 nm), submicrometer (100 nm to 1 μm), subvisible (1 μm to 100 μm), and visible (> 100 μm). However, the boundary between subvisible and visible particles is not absolute, as detectability by the naked eye depends on visual factors such as particle quantity, size, shape, and density^13^. VPs can vary among therapeutic protein DPs due to differences in formulation and manufacturing processes, necessitating tailored risk assessments, particle source identification, and selection of effective analytical methods^14^.

According to USP < 1790>, visual inspections can be conducted using manual, semi-automated, or automated methods. Manual inspection involves an inspector examining each product by hand against a solid-colored backdrop under adequate lighting. Semi-automated inspection uses mechanical assistance, rotating the product at a set rate in front of the inspector. Automated inspection, which can supplement or replace manual methods, relies on techniques such as light obscuration or electronic image analysis to detect VPs^13^.

Manual and semi-automated techniques depend on the human eye, potentially introducing variability and subjectivity in VP detection. Automated methods, such as light obscuration used for subvisible particle detection, often significantly underestimate particle concentration and size^15–17^. More recently, flow-imaging (FI) based methods mostly used for subvisible particle detection have been used for VP detection. Flow imaging methods offer higher throughput, increased particle detection sensitivity, and morphological data, but lack the capability to perform chemical identification, which is helpful for root cause analysis (RCA) and building visual particulate libraries. Identifying particulate sources is necessary in RCA investigations to help prevent future instances of particulate formation. Because particle formation in therapeutic protein DPs can arise from a multitude of mechanisms influenced by formulation, storage, and handling, RCA often uses a combination of techniques for a comprehensive investigation and proper mitigation^18^.

Current industry practice in developing procedures for detecting and characterizing VPs involves suitable standards like polystyrene microspheres for validation. Polystyrene (PS) microspheres are monodisperse, spherical particle standards with high optical contrast and are commonly used for particle characterization analytical procedure validation^19,20^. However, PS differs from proteinaceous VPs found in therapeutic proteins, which typically have low optical contrast and irregular morphologies^21^. To address these limitations, the US National Institute of Standards and Technology (NIST) developed visible protein-like particle surrogates to mimic the appearance of proteinaceous particles^22,23^. These surrogates include photolithographic, monodisperse particles fabricated from SU-8 photoresist and polydisperse ethylene tetrafluoroethylene (ETFE) particles. ETFE particles are non-spherical and have low optical contrast. SU-8 particles have a non-spherical morphology and are largely transparent when slide mounted, even though they have a high refractive index. These candidate materials can allow for the validation of more advanced particle detection and characterization methods in therapeutic protein DPs.

Morphologically Directed Raman Spectroscopy (MDRS) has been previously used to analyze illicit and counterfeit drug samples^24^, demonstrate bioequivalence in nasal sprays^25^, and characterize subvisible particulates in therapeutic proteins^26^. This technique combines automated image analysis to extract particulate morphological data with Raman spectroscopy for chemical identification. Unlike traditional Raman spectroscopic microscopy, which is commonly used for intracellular or surface chemical analysis and typically involves manual selection of regions of interest^27^, MDRS enables automated analysis of particles based on their morphology. This work explores the utility of MDRS in detecting and characterizing VPs in therapeutic protein DPs to support RCA investigations, along with assessing the suitability of candidate reference materials.

## Results

### Morphological analysis of monodisperse visible particles

**Fig. 1:**
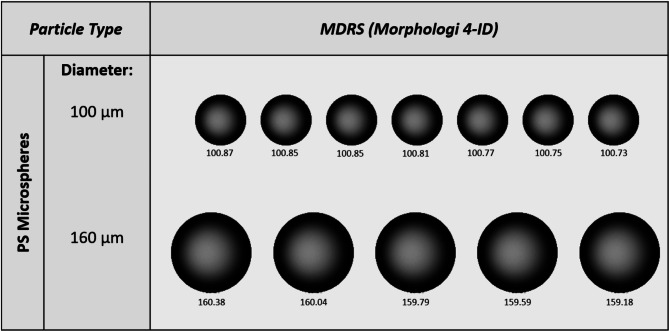
Representative images of PS microspheres larger than 100 μm captured by MDRS.

**Fig. 2:**
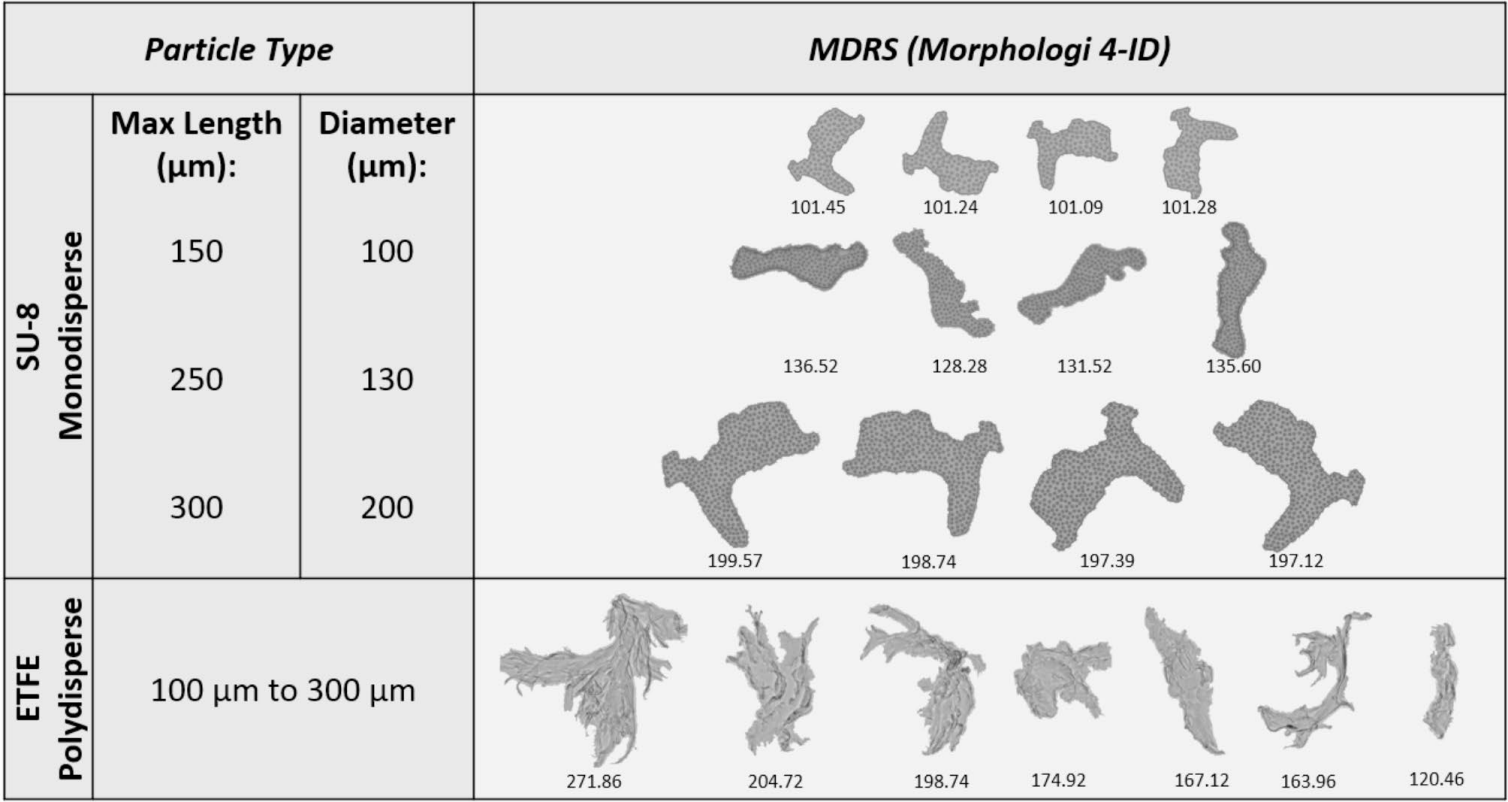
Representative images of NIST Standards: SU-8 (150 μm, 250 μm, & 300 μm) and ETFE (106 & 150 μm) captured by MDRS.

Industry-grade monodisperse particle standards were analyzed to evaluate the size and shape measurement capabilities of MDRS. First, different sizes of PS microspheres were compared to manufacturer’s specified sizes for accuracy (Fig. 1). For 160 μm and 100 μm microspheres, 50 and 73 particles were detected and analyzed, respectively. The Morphologi 4-ID software slightly overestimated the mean diameter compared to the specifications, while standard deviations remained comparable. The specification diameters were 160 μm ± 2.9 μm SD for 160 μm microspheres and 100 μm ± 1.6 μm SD for 100 μm microspheres, and the measured diameters were 163 ± 3.0 μm SD for the 160 μm microspheres and 101 ± 1.1 μm SD for the 100 μm microspheres. All images of PS microspheres were easily focused and demonstrated their uniform size. Next, NIST SU-8 particles were measured for size (Fig. 2). SU-8 particles in this study were defined by the diameter of the largest circle that circumscribes that particle, equivalent to the maximum length in micrometers^23^. For SU-8 300 μm, 250 μm, and 150 μm particles, 17, 12, and 26 VPs were detected and analyzed, respectively. Maximum length and aspect ratio measurements from MDRS were generally in line with NIST values (not reference values) obtained from flow imaging measurements^23^, as summarized in Table 1. SU-8 particles displayed well-defined shapes and were easy to focus during imaging.

**Table 1:** Morphological measurements of SU-8 particles.

NIST max length (µm)	MDRS max length (µm)	NIST aspect ratio	MDRS aspect ratio
150 µm	152 ± 1.7	0.62 ± 0.02	0.84 ± 0.05
250 µm	257 ± 1.5	0.27 ± 0.01	0.35 ± 0.01
300 µm	300 ± 1.0	0.61 ± 0.01	0.81 ± 0.08

Error bars represent standard deviation. Definitions for maximum length and aspect ratio are included in Table S1.

### Morphological analysis of polydisperse VP candidate reference material

Polydisperse ETFE VPs were analyzed to evaluate the size and shape measurement capabilities of MDRS and their potential use as protein-like particle surrogates (Fig. 2). A total of 16 ETFE particles were detected and analyzed, with 12 from the ES150 sample and 4 from the ES106 sample. According to Telikepalli et al.^23^, ETFE ES150 particles are typically enriched in the 150 μm to 250 μm diameter range, while ES106 particles are enriched in the 106 μm to 150 μm range. MDRS detected particle diameters (equivalent spherical diameter, ESD) ranging from 110 μm to 272 μm for ES150 and 106 μm to 122 μm for ES106.

### Morphological analysis of stressed therapeutic protein visible particles

**Fig. 3:**
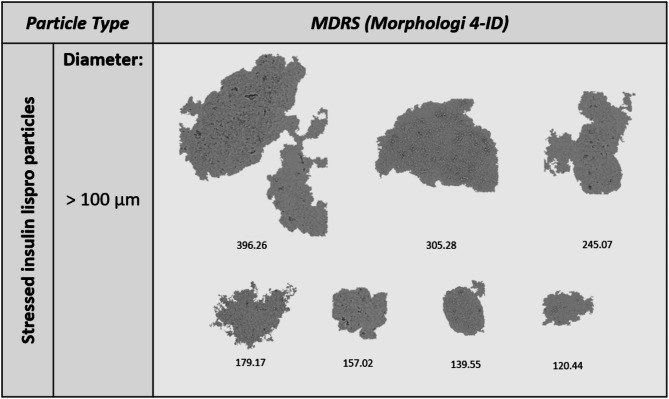
Representative images of visible particles formed in stressed insulin lispro subjected to 300 rpm for 2 h at 70 °C.

**Fig. 4:**
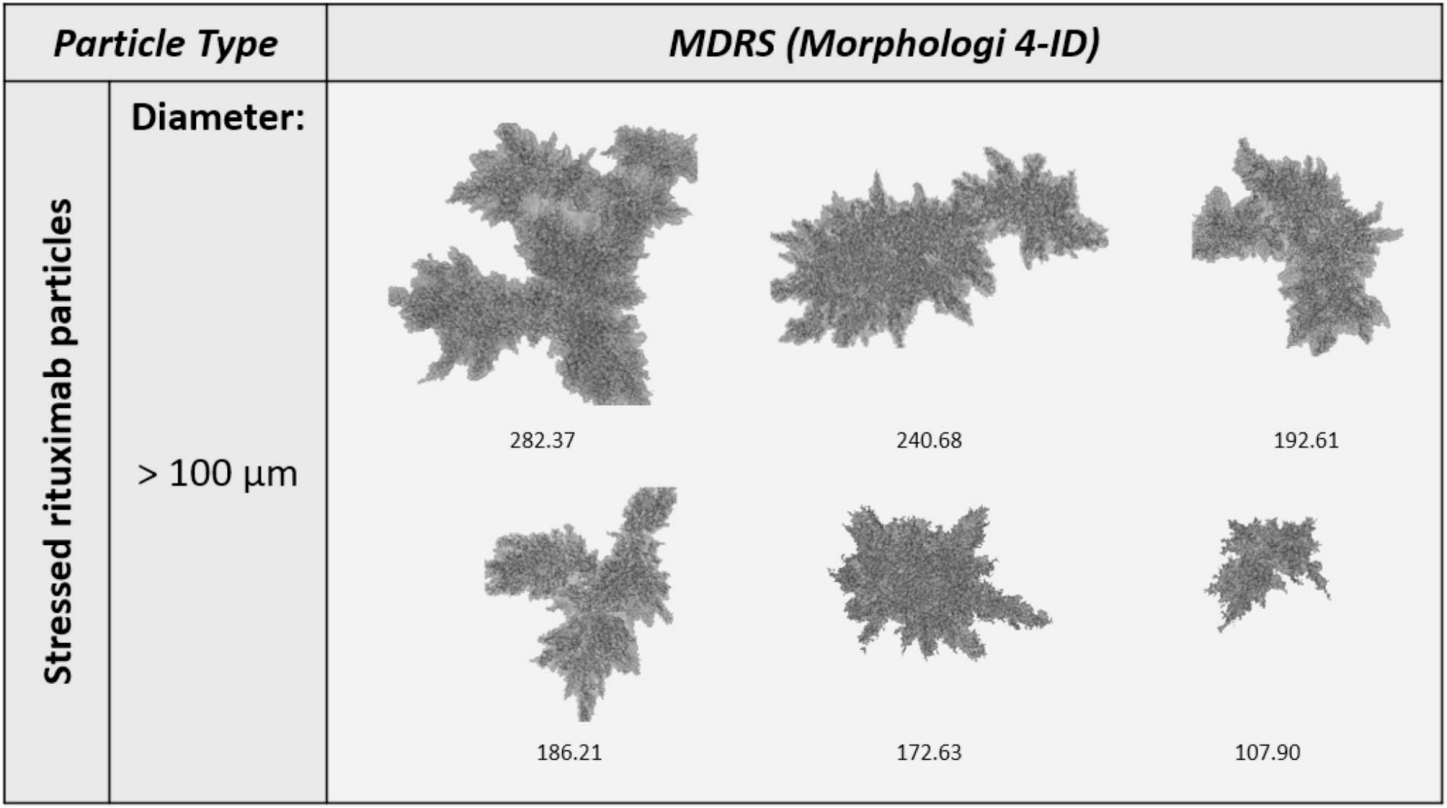
Representative images of visible particles formed in stressed rituximab subjected to 300 rpm for 2 h at 70 °C.

Model stressed therapeutic protein DPs were analyzed using MDRS. Insulin lispro and rituximab were subjected to thermo-mechanical stress to generate VPs, outlined in the “Methods and Materials” section. All particles larger than 100 μm were morphologically analyzed. A total of 197 insulin lispro and 67 rituximab visible particles were detected. Insulin lispro yielded more transparent, film-like particles with less defined borders (Fig. 3). In contrast, the rituximab sample yielded more jagged, irregular particles with less translucency (Fig. 4).

### Principal component analysis of VP morphological features

**Fig. 5:**
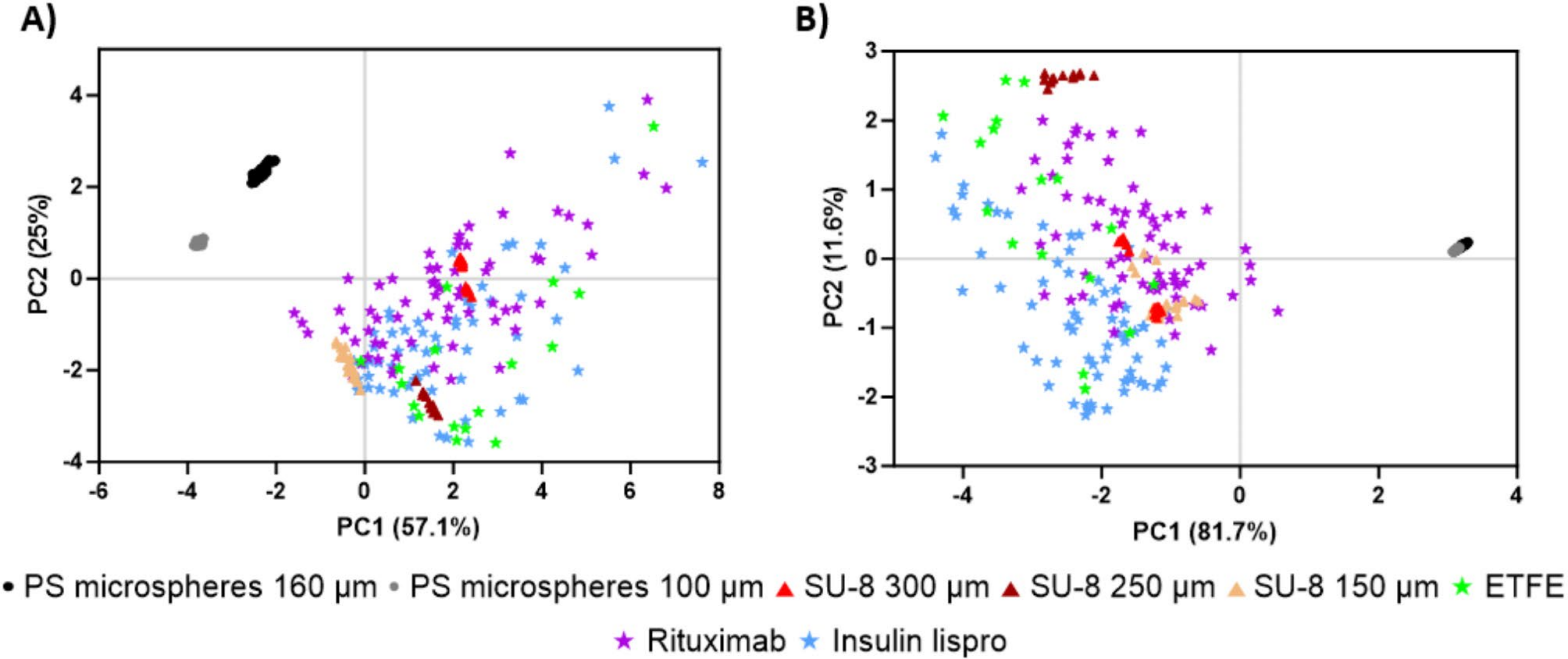
Composite morphological score using principal component analysis (PCA) of morphological features (Table S1) for VP standards, NIST candidate reference materials, and stressed TPs. **(A)** PCA with all features (size, shape, transparency). **(B)** PCA with only shape and transparency features. Each point represents one particle. Results show tight clustering for PS microspheres and SU-8 particles, compared to dispersion throughout the plot for ETFE particles and stressed TP VPs.

A composite morphological score for each VP group was generated using principal component analysis (PCA), which incorporated morphological features measured by Morphologi-4 ID (Table 2). PCA is a statistical tool that can be used to reduce the dimensionality of complex datasets to simplify identification of patterns in data^28^. PCA has been used in a wide range of biotechnology applications, including cell morphological categorization^29^, nanoparticle categorization^30^, and analyzing drug granule properties^31^. In this work, PCA was applied using all morphological features measured by the instrument software—a total of sixteen features encompassing particle size, shape, and transparency (Table 2). The analysis was first conducted using all sixteen features (Fig. 5A) and then repeated with features #9–16 to focus on shape and transparency features alone (Fig. 5B). The same number of insulin lispro and rituximab VPs (67 each) were included in the PCA, with all the detected rituximab VPs and a representative selection of insulin lispro VPs based on the diameter distribution of the full set of 197 particles. In both PCA plots, monodisperse VPs (PS microspheres, SU-8) formed tightly clustered groups. In the first PCA plot, PC1 captured 57.1% of variance in the data set, with PC2 capturing 25%. PS microspheres clustered more negatively along the PC1 axis and were separated into two clusters based on sphere diameter. Similarly, SU-8 particles formed tight clusters that were also size-dependent. Conversely, ETFE particles exhibited a broader distribution across the plot, indicating greater morphological variability. Similarly stressed therapeutic protein VPs exhibited a wider spread across both PCA plots, akin to ETFE, further highlighting the morphological diversity of these VPs (Fig. 5A). PCA conducted using all sixteen features was influenced by size parameters (e.g., area, diameter), as VPs with similar shapes did not cluster together. To better assess the suitability of candidate standards based on protein-like characteristics, a second PCA was performed with features #9–16, which excluded size features, to offer more insights into morphological variability. A more negative PC1 score corresponded to more transparent, film-like particles, while increasing positivity along PC1 indicated more opaque, compact particles. PC2 captured variations in aspect ratio, with lower aspect ratios corresponding to more positive values along the axis. In the follow-up PCA, PC1 captured 81.7% of variance in the data set and PC2 captured 11.6%. In the focused PCA analysis, monodisperse VPs formed tight clusters, as was observed in the PCA using all features, with their shape and transparency features better represented. PS microspheres from both diameters formed a single cluster, and SU-8 particles (150 μm and 300 μm maximum length) clustered together. ETFE’s morphological variability became more apparent in the size-excluded PCA, with stressed therapeutic protein particles displaying similar characteristics, scattering widely throughout the plot. SU-8 250 μm exhibited a higher position on PC2 which was attributed to lower aspect ratio, while PS microspheres remained near 0, reflecting their inherently spherical morphology (Fig. 5B). PCA plots were generated excluding stressed therapeutic protein VPs for improved visualization of standards and candidate reference materials (Fig. S1). Detailed histograms of morphological features’ aspect ratio, circularity, convexity, and elongation further illustrate the limited morphological representation of polystyrene microspheres and SU-8 compared to ETFE (Fig. S2), highlighting ETFE’s similarities to stressed therapeutic protein VPs (Fig. S3).

**Table 2:**
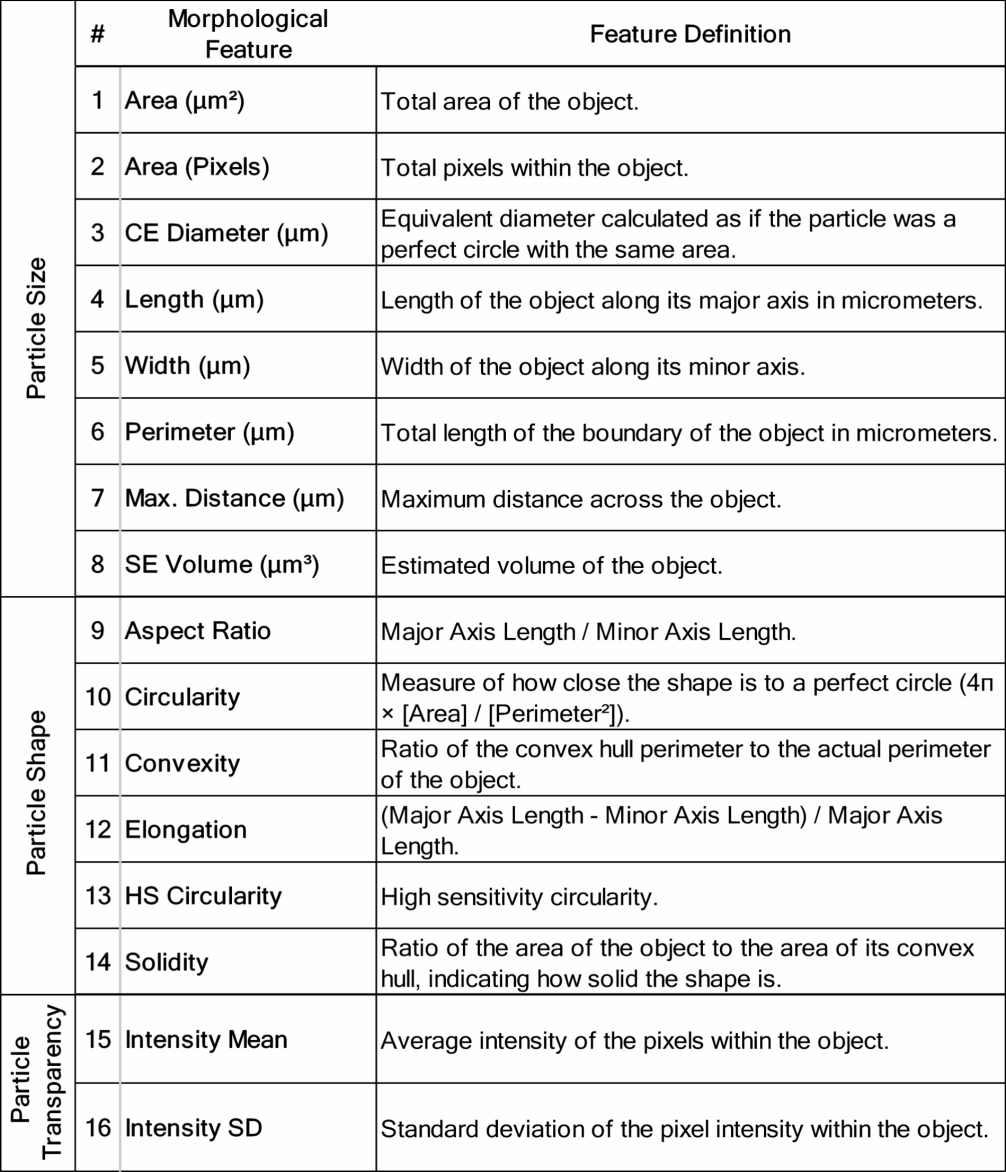
Summary of morphological features measured by MDRS and their descriptions.

### Chemical identification of VPs using Raman spectroscopy

**Fig. 6:**
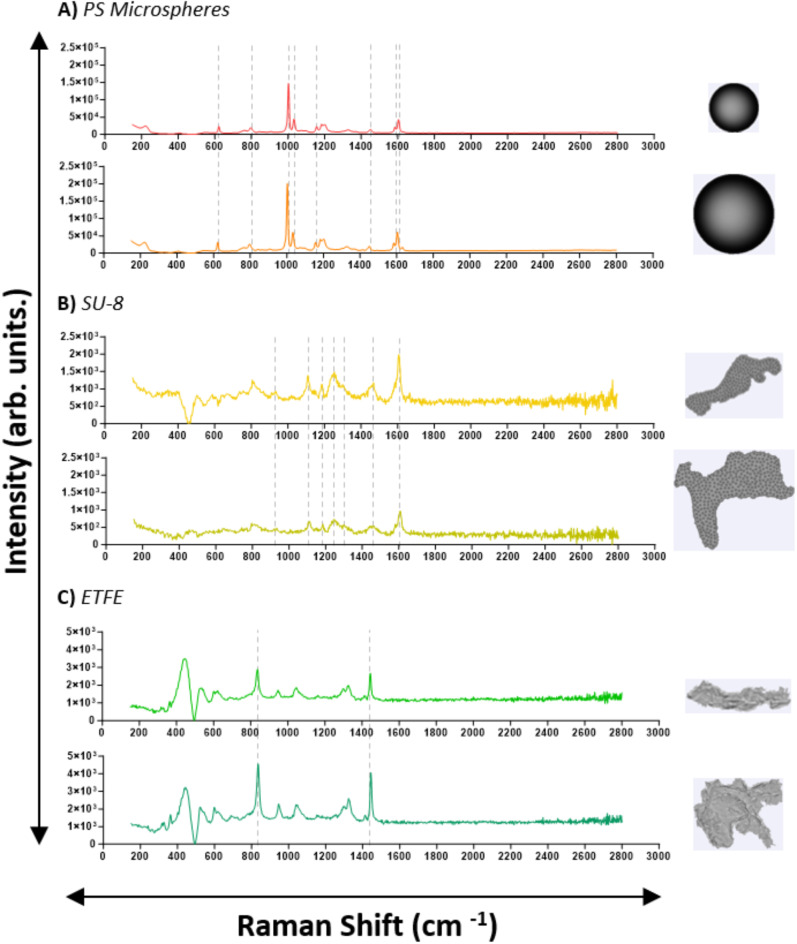
Representative Raman spectra of **(A)** PS microspheres, **(B)** SU-8 particles, and **(C)** ETFE particles. Characteristic peaks (Table 3) are highlighted with grey dashed lines.

**Fig. 7:**
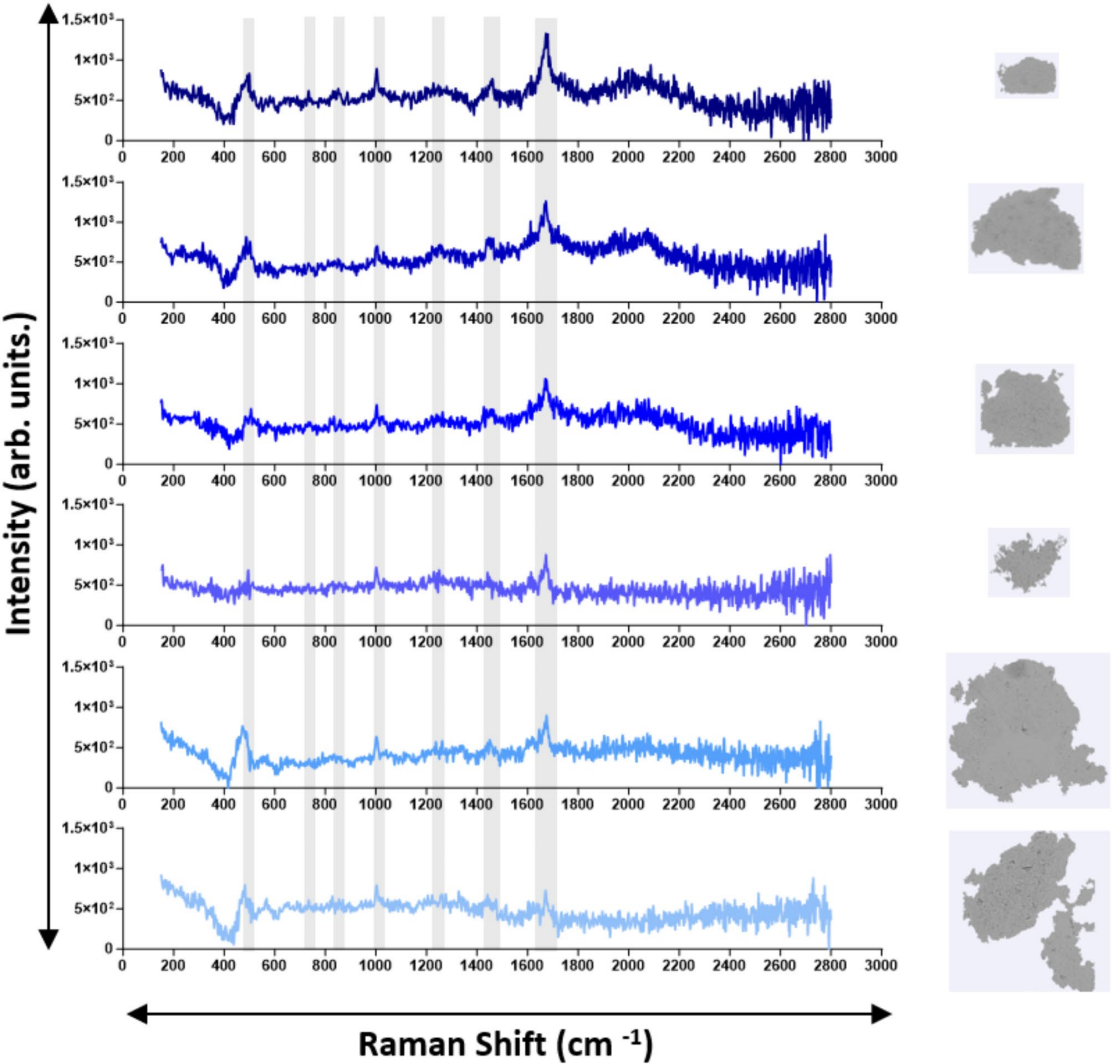
Representative Raman spectra of VPs found in stressed insulin lispro. Characteristic proteinaceous peaks (Table 3) are indicated in grey shaded regions.

**Fig. 8:**
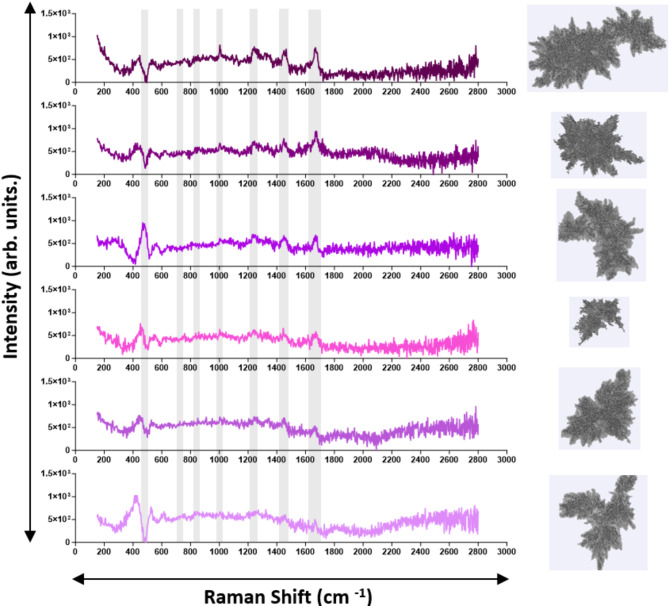
Representative Raman spectra of VPs found in stressed rituximab. Characteristic proteinaceous peaks (Table 4) are indicated in grey shaded regions.

We collected Raman spectra for all particle types (PS microspheres SU-8, ETFE) and stressed therapeutic proteins to confirm their chemical identity. Both 100 μm and 160 μm diameter PS microspheres exhibited 8 sharp, characteristic polystyrene peaks, with intensity increasing with size^32,33^ SU-8 particles displayed 7 sharp characteristic peaks^34,35^, while ETFE showed 2 characteristic peaks^36^ (Fig. 6). A detailed description of their Raman shifts and corresponding assignment is provided in Table 3. Raman spectra were also collected for stressed insulin lispro. Insulin VPs exhibited characteristic proteinaceous peaks^37–39^, though their intensities varied between 750 and 1500 arb. units., depending on optical contrast, with more transparent, film-like particles showing lower intensities and reduced sharpness (Fig. 7). Stressed rituximab particles also demonstrated well-defined proteinaceous peaks^40^ (Fig. 8), with varying intensities between 500 and 1000 arb. units. A detailed description of protein Raman shifts and their corresponding assignments is provided in Table 4. For Figs. 6 and 7, the background spectrum of the quartz glass microscope slide exhibited a broad peak between ~ 300–550 cm⁻¹ and a relatively constant baseline at higher frequencies. After subtracting this background, the particle spectrum in the lower frequency region (~ 300–550 cm⁻¹) may have dipped below the baseline observed at higher frequencies due to the removal of the quartz glass contribution.

**Table 3:**
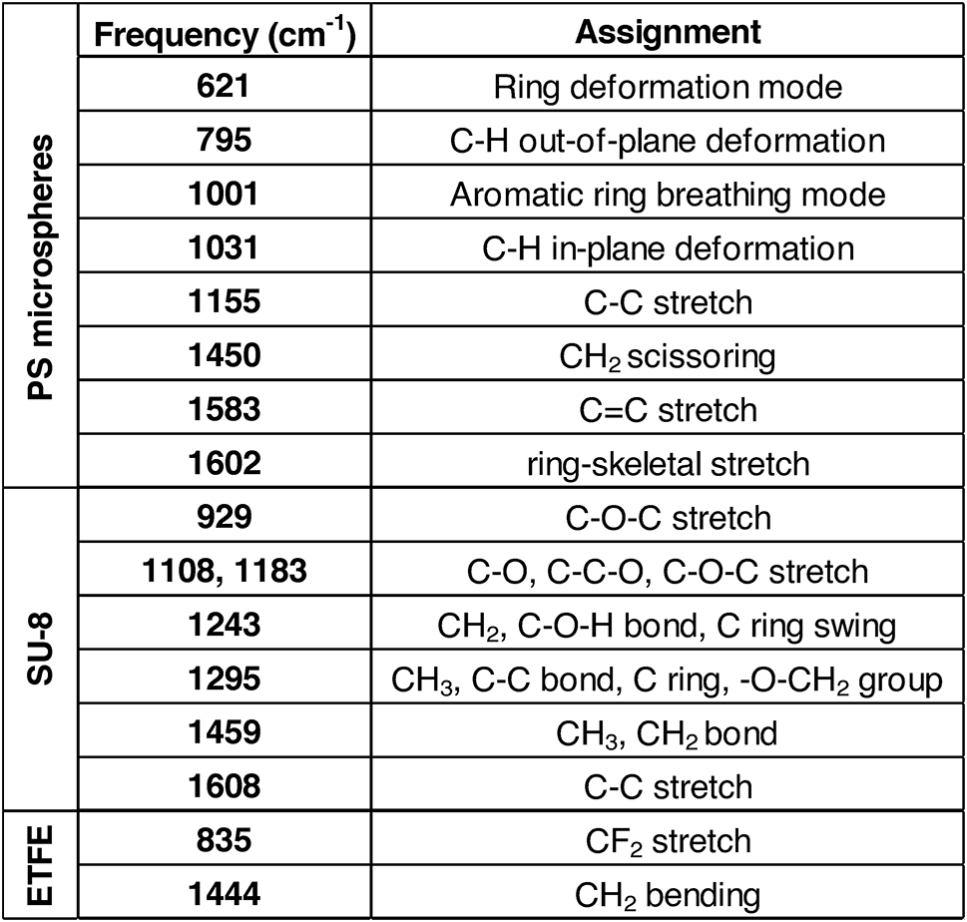
Characteristic Raman shifts of PS microspheres, SU-8, and ETFE standards.

**Table 4:**
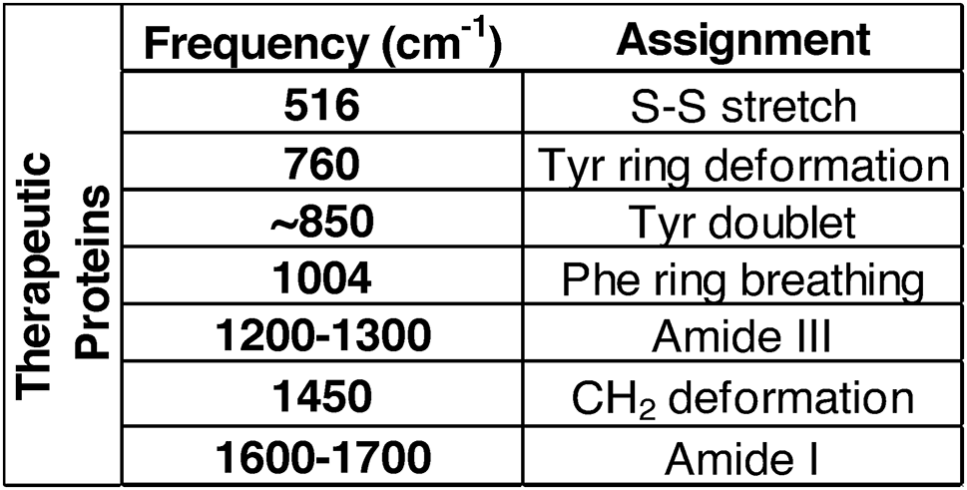
Characteristic Raman shifts of proteinaceous aggregates.

## Discussion

VP contamination is one of the leading causes of injectable product recalls^41^. Since 2018, 22 drug product recalls have occurred due to particulates, including glass^42,43^, silicone^44,45^, proteinaceous material^46^, and unidentified materials^47^. These cases underscore the need for robust RCA tools. The agency has received manufacturer reports of cellulose VPs that were only detected during stability testing because of insufficient analytical procedure detection sensitivity for control of VPs during release testing. Atypical fiber VPs, ranging from 96 μm to 2000 μm in length, were also detected during vial defect classification. Elemental composition and morphological analysis indicated that material from filters used in processing was a potential source of these particles. In another submission, the reconstitution of a therapeutic protein drug product revealed black particles in the solution. Analytical characterization determined that these particles were heat-exposed, partially oxidized, melted, and charred polyamide derivatives. Upon removal of the vial stopper, similar particles were observed between the stopper and the inner surface of the glass vial neck. These examples underscore the importance of developing improved tools to support RCA by identifying potential sources of VPs, emphasizing that multiple factors can contribute to their presence. A method capable of providing well-resolved morphological and parametric analysis would be invaluable in addressing such drug product quality issues.

Several compendial VP detection methods rely on subjective visual inspection, which can be supplemented by techniques such as Fourier Transform Infrared Spectroscopy (FTIR) or Raman spectroscopy for chemical identification^48,49^. In this study, we applied a combined approach for VP detection and characterization that integrated morphological assessment with chemical identification in a single, efficient tool. The approach also includes the use of PCA to compare the morphological characteristics of the various VP types. Our data demonstrated that the method successfully differentiated between monodisperse particles (PS microspheres, SU-8), polydisperse particles (ETFE), and stressed therapeutic protein DPs (insulin lispro and rituximab). Importantly, the capability to identify different particle types can allow for the implementation of tailored measures to mitigate their presence.

High-resolution images of PS microspheres (Fig. 1) provided diameters closely matching manufacturer specifications. Similarly, images of NIST SU-8 candidate reference material (Fig. 2) recorded morphological measurements (e.g., aspect ratio, diameter) that were consistent with values provided by NIST. These findings highlight the utility of high-resolution morphological analysis techniques for accurately characterizing particle dimensions and shapes. We demonstrated this in our method using both established reference standards and novel candidate materials.

It is known that protein aggregates can display a wide range of morphologies^50^, a characteristic not accurately captured by monodisperse VP standards. Accurate detection and characterization of inherent proteinaceous VPs are important for ensuring the quality of therapeutic proteins. The need for identification and characterization led to the development of ETFE protein-like particle surrogates at NIST for use in formulation and process development of biopharmaceuticals^22^. In the assessment of NIST ETFE particles, high-resolution imaging captured VPs across a range of sizes and shapes (Fig. 2). These results demonstrated the capability of high-resolution morphological analysis to detect VPs with diverse morphologies, supporting the potential use of ETFE particles as candidate reference material for visual inspection training purposes^23^.

We applied our MDRS method to marketed model therapeutic protein drugs, including insulin lispro and rituximab, to evaluate the effectiveness of the technique on real drug products and compare therapeutic protein VP morphologies to those of the NIST ETFE. To induce VP formation, therapeutic proteins were subjected to extreme thermo-mechanical stress conditions. The resulting VPs differed between DP class, with insulin lispro presenting more film-like, transparent morphologies (Fig. 3) and rituximab with more amorphous, complex morphologies (Fig. 4). Previous studies have shown that protein aggregate morphology changes under different stress conditions in insulin and mAb products^51,52^, and these differences may also be influenced by formulation variations. Although MDRS detected thousands of particles in the stressed therapeutic proteins, less than 1% were greater than 100 μm, which was the primary focus of this study.

PCA was employed in this study to compare morphological properties of VP reference material against VPs from stressed therapeutic proteins. PCA was conducted using sixteen morphological features measured by MDRS (Table 2) and demonstrated tight clustering in monodisperse VPs (PS microspheres, SU-8), whereas ETFE and stressed therapeutic proteins VPs exhibited more scattering throughout the PCA plot (Fig. 5A). The results underscored the suitability of ETFE as a protein surrogate due to its morphological diversity, while highlighting the limitations of monodisperse VP standards in representing the wide range of morphologies seen in protein aggregates. To further visualize the suitability of ETFE, another PCA was run excluding 8 size features (Fig. 5B) to emphasize protein-like characteristics such as irregular shape and low optical contrast. Here, we observed a dispersion of ETFE throughout the PCA plot, showing a pattern like that observed for stressed therapeutic proteins. In contrast, monodisperse particles, particularly PS microspheres, remained tightly clustered far from other groups, further emphasizing the limited variability in their morphology. PCA analysis of morphology data offered valuable insights into the suitability of candidate reference materials for enhancing therapeutic protein DP quality control. By distinguishing among morphological properties, this approach supports the selection of reference materials that better represent the diverse characteristics of protein aggregates.

Immediate chemical characterization of VPs following morphological imaging is valuable for RCA applications, as such data enables real-time identification of chemical compositions and enhances the efficiency of investigations into VP contamination in therapeutic proteins. We first tested the application of MDRS on candidate VP reference materials, and the Raman spectra obtained for each exhibited sharp and distinct characteristic peaks, confirming their chemical identity (Fig. 6; Table 3). Raman spectra were then obtained from VPs from stressed insulin (Fig. 6) and mAb (Fig. 7) samples. All spectra exhibited signature proteinaceous peaks (Table 4), with their intensities seemingly varying with the complexity of the VP. In general, rituximab exhibited less intense peaks than insulin lispro, at around 1000 arb. units. compared to 1500 arb. units. for insulin lispro. Rituximab showed more pronounced peaks in the 1200 to 1300 cm^−1^ (amide III) and 1450 cm^−1^ (CH_2_ deformation) regions, but most VPs had the absence of the 516 cm^− 1^ peak (S-S stretch) that was observed in the insulin spectra. These variations indicated that MDRS could detect sufficient differences in spectra, to provide deeper insights into the structural properties and interactions of VPs within the formulation and further contribute to RCA investigations.

## Conclusion

The presence of VPs in therapeutic protein DPs remains a challenge for the pharmaceutical industry, with RCA playing a key role in addressing this product quality issue. The data from this study demonstrated an effective approach for detecting and identifying VPs in two different classes of therapeutic protein DPs (i.e., insulin, mAbs) by combining morphological measurements with chemical identification. This integrated methodology could enable manufacturers to trace particulate sources and mitigate their presence. Our method was tested using industry standards (PS microspheres), NIST candidate reference materials (SU-8 and ETFE) and stressed therapeutic protein VPs. High-quality images of VPs and morphological measurements were obtained, along with Raman spectra that confirmed their chemical identities. PCA of morphological features revealed that the NIST ETFE particles exhibited similar morphological variation to VPs found in stressed therapeutic protein DPs, supporting the use of NIST ETFE as a protein-like particle surrogate for visual inspection training. This MDRS methodology has the potential to contribute to a comprehensive database of particle morphology and chemical composition to improve particle classification and facilitate more effective RCA investigations. Overall, this integrated approach demonstrated promise in enhancing particle detection and characterization efforts, contributing to the development of high-quality therapeutic protein DPs.

## Methods and materials

### Sample preparation

Polystyrene microsphere standards (100 μm and 160 μm diameter) were purchased from Duke Scientific through Thermo Fisher Scientific.

SU-8 particles were made as described by Telikepalli et al.,^23^ in the NIST NanoFab. The particles were fabricated on silicon wafers by first coating the wafers with a sacrificial Omnicoat release layer. SU-8 2005 resin was then spun onto the wafers at controlled speeds to achieve the desired particle thickness. After soft baking the resin, the wafers were patterned and exposed using a high-resolution contactless aligner tool, followed by a post-exposure bake to crosslink the SU-8 polymer. The non-crosslinked SU-8 was removed, and the wafer was plasma etched to eliminate any residue. To release the particles, the wafer was submerged in a solution of Remover 1165 and Triton X-100, which dissolved the Omnicoat layer and coated the particles with the Triton surfactant. The particles were then filtered, washed with isopropanol, and suspended in a Triton-containing diluent. SU-8 particles are labeled based on their maximum length in microns (i.e., 150, 250, or 300 μm).

ETFE particles were made as described by Telikepalli et al.,^23^. The ETFE particles were prepared using a modified milling machine. An ETFE rod was milled using a carbide burr with a diamond tooth pattern to produce irregular particles. These particles were collected in a diluent containing Triton X-305, Triton X-100, and sodium azide in deionized ultra-filtered water. After milling, the ETFE particles were filtered and enriched in size fractions using nylon mesh sieves. The filtered particles were then washed, agitated, and stored at 2 to 8 °C. Different size fractions, such as ES150, ES106, and ES75, were collected based on the sieve used during filtration, with particles enriched in the desired size range. ETFE particles are labeled “ES” followed by the sieve size in microns used for enrichment of certain particle sizes^23^.

Insulin and monoclonal antibody (mAb) DPs were used as model stressed therapeutic proteins DPs for this study. Insulin lispro (Humalog, Eli Lilly, Indianapolis, IN, USA) and rituximab (Rituxan, Genentech, South San Francisco, CA, USA) were sourced commercially. 2 mL of insulin lispro and 2 mL of rituximab were aliquoted into glass vials and exposed to combined thermo-mechanical stress conditions. Samples were placed on a benchtop incubator-shaker (Thermo Scientific, MaxQ 4450) at 70 °C and 300 rpm for 2 h. Following stress exposure, the insulin and mAb samples were analyzed.

### Morphologically directed Raman spectroscopy (MDRS)

Morphologi 4-ID (Malvern Panalytical Ltd), which integrates automated particle imaging with Raman spectroscopy, was utilized to obtain morphological characteristics, particle counts, and chemical identification of VPs. An aliquot of 20 µL of sample was pipetted onto a quartz glass microscope slide (size: 25 × 75 mm) and covered with a square quartz glass coverslip (size: 19 × 19 mm) to ensure even sample dispersion. The perimeter of the coverslip was sealed with nail polish to prevent evaporation, and the slide was left undisturbed for 30 min to allow particles to settle. The instrument offers five magnifications (2.5 X, 5 X, 10 X, 20 X, and 50 X). Particles were imaged using the automated ‘Sharp Edge’ segmentation analysis setting at 10 X magnification, covering a resolution range of 2.5 to 160 μm, to obtain morphological data. After imaging within the specified area, particles of interest were selected for chemical identification using a 20 s exposure time at high laser power. Raman spectra were acquired at 50 X magnification, with a resolution of 0.5 to 50 μm, and a 2 μm laser spot size. All particles greater than 100 μm in the stressed therapeutic protein samples were included for morphological analysis. Morphological data from all VPs were analyzed using principal component analysis (PCA) in JMP Pro 17, based on correlations among sixteen particle size, shape, and transparency features. A comprehensive description of all morphological features is provided in Table 2. Features were collected using Morphologi 4-ID software version 10.20 (https://www.malvernpanalytical.com/en/support/product-support/morphologi-range/morphologi-4-id) and include specific parameters such as particle diameter, aspect ratio, circularity, and transparency, among others.

## Electronic supplementary material

Below is the link to the electronic supplementary material.

Supplementary Material 1

## Data Availability

The datasets generated during and/or analyzed during the current study are available from the corresponding author on reasonable request.

## Abbreviations

DP: Drug product
ES: ETFE size
ETFE: Ethylene tetrafluoroethylene
FTIR: Fourier transform infrared spectroscopy
MDRS: Morphologically directed Raman spectroscopy
mAb: Monoclonal antibody
NIST: National Institutes of Standards and Technology
PCA: Principal component analysis
PC: Principal component
PS: Polystyrene
RCA: Root cause analysis
USP: United States Pharmacopeia

## Declarations

### Disclaimer

This manuscript reflects the views of the author and should not be construed to represent FDA’s or NIST’s views or policies. Certain commercial equipment, instruments, or materials are identified in this manuscript to foster understanding. Such identification does not imply recommendation or endorsement by FDA or the National Institute of Standards and Technology, nor does it imply that the materials or equipment identified are necessarily the best available for the purpose. This work was carried out in part in the NIST Center for Nanoscale Science and Technology NanoFab.
