# Supplementary material for: Root cause analysis investigation of visible particulates in therapeutic protein drug products using morphologically directed Raman spectroscopy
Source: Sci Rep. 2025 Nov 26;15:42026. doi: 10.1038/s41598-025-97097-x (PMC12657923; doi:10.1038/s41598-025-97097-x)
Supplement: Supplementary file 1 — Supplementary Material 1 [file 41598_2025_97097_MOESM1_ESM.pdf]

# Root Cause Analysis Investigation of Visible Particulates in Therapeutic Protein Drug Products using Morphologically Directed Raman Spectroscopy

**Isabella F de Luna[1], Srivalli N Telikepalli[2], Michael Carrier[2], Dean Ripple[3], Charudharshini Srinivasan[1§], Thomas O'Connor[1], Scott Lute[1], Ashwinkumar Bhirde[1*]**

[1]Division of Pharmaceutical Quality Research VI, Office of Pharmaceutical Quality Research,

[1§]Division of Product Quality Assessment II (DPQAII), Office of Product Quality Assessment (OPQA I),

Office of Pharmaceutical Quality, Center for Drug Evaluation and Research, Food and Drug Administration, Silver Spring, MD, USA.

[2]Biomolecular Measurement Division, National Institute of Standards and Technology, Gaithersburg, MD, USA

[3]Biomolecular Measurement Division, National Institute of Standards and Technology, Gaithersburg, MD, USA (retired)

*Corresponding author contact information: Ashwinkumar.Bhirde@hhs.fda.gov

**SUPPLEMENTARY FIGURES**

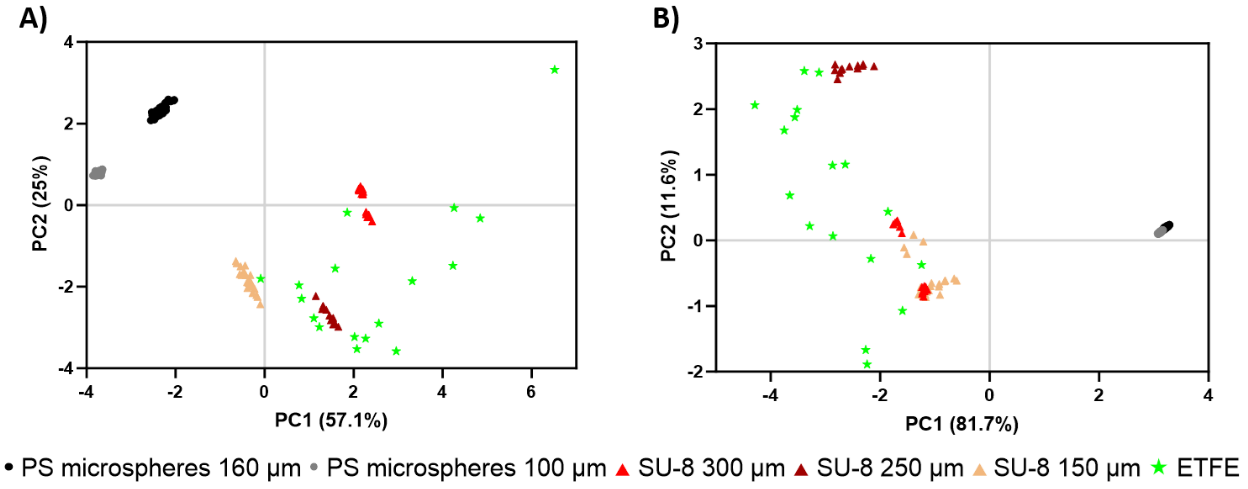


**Supplementary Fig. S1.** Composite morphological score using principal component analysis (PCA) of morphological features (Table S1) for VP standards and NIST candidate reference material. A. PCA with all features (size, shape, transparency). B. PCA with only shape and transparency features. Each point represents one particle. Results show tight clustering for PS beads and SU-8 particles, compared to dispersion throughout the plot for ETFE particles.

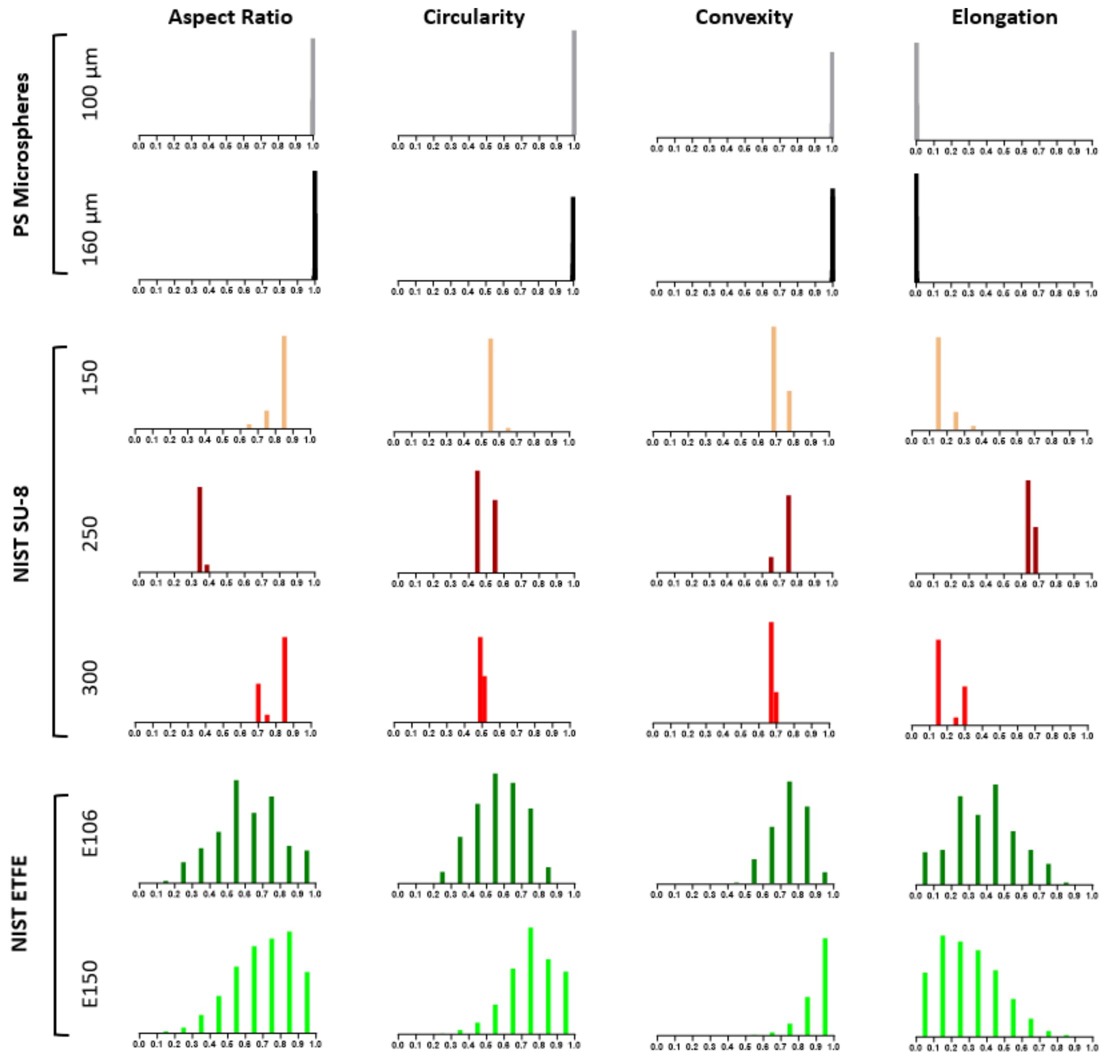


**Supplementary Fig. S2.** Morphological histograms of VP standards and NIST candidate reference materials. Aspect ratio, circularity, convexity, and elongation histograms were compared.

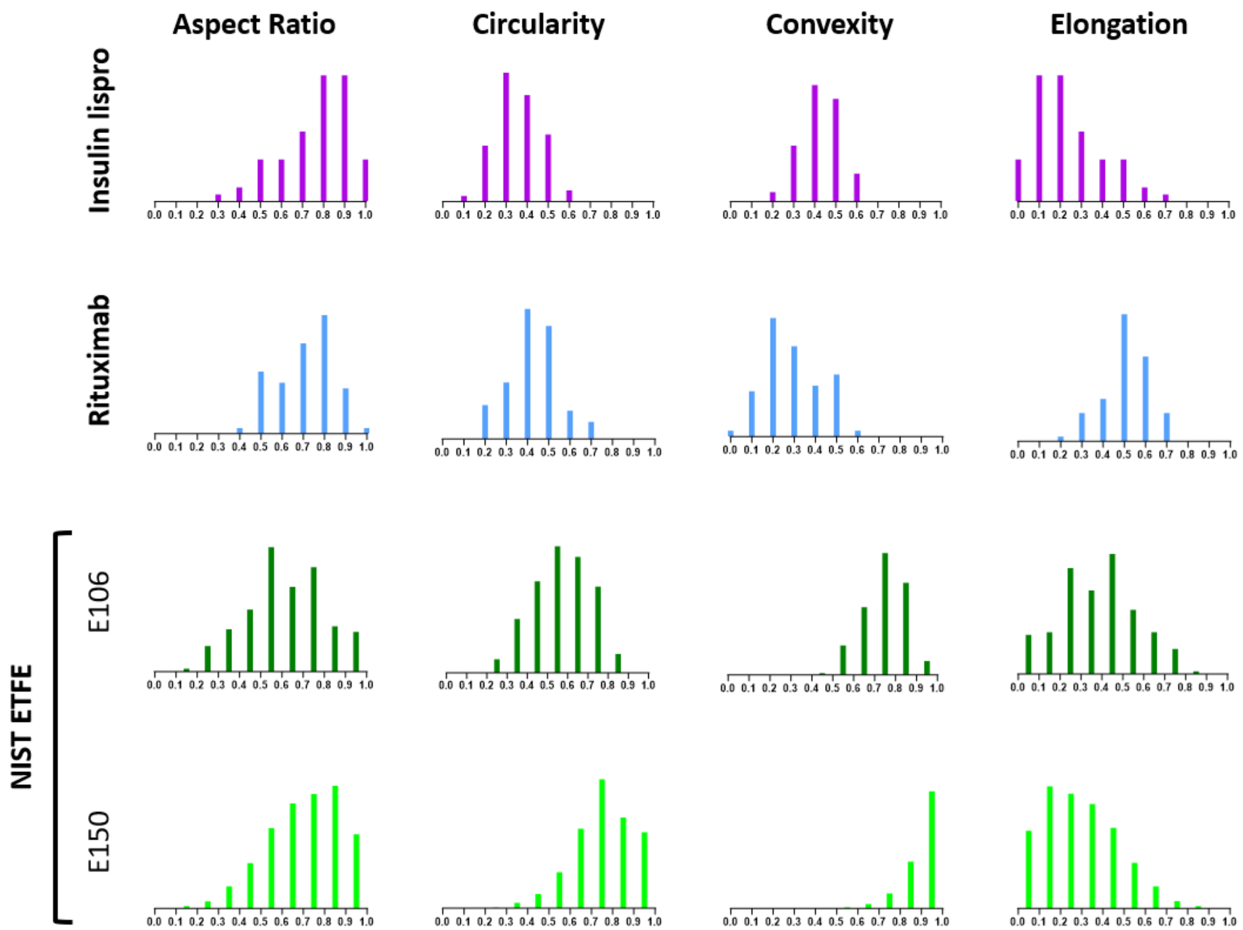


**Supplementary Fig. S3.** Morphological histograms of stressed TP VPs and ETFE. Aspect ratio, circularity, convexity, and elongation histograms were compared.
